# Polyamine uptake in the malaria parasite, *Plasmodium falciparum,* is dependent on the parasite's membrane potential

**DOI:** 10.1186/1475-2875-9-S2-O24

**Published:** 2010-10-20

**Authors:** J Niemand, L Birkholtz, AI Louw, K Kirk

**Affiliations:** 1 Department of Biochemistry, Faculty of Natural and Agricultural Sciences, University of Pretoria, Pretoria, South Africa 0002; 2 Research School of Biology, Australian National University, Canberra, ACT, Australia 0200

## 

Polyamines are present at high levels in proliferating cells, including cancerous cells and protozoan parasites and the inhibition of their synthesis has been exploited in antiproliferative strategies. Inhibition of the malaria parasite's polyamine biosynthetic pathway causes cytostatic arrest in the trophozoite stage but does not cure *in vivo* infections in the murine model of malaria. This is possibly due to exogenous polyamine salvage from the host, which replenishes the intracellular polyamine pool.

This implies that disruption of polyamine metabolism as an antimalarial chemotherapy strategy may require targeting both polyamine biosynthesis and transport simultaneously.

In the absence of a clear understanding of the uptake mechanism of polyamines into *Plasmodium falciparum* parasites, polyamine transport into both the infected erythrocytes and parasites isolated from the erythrocyte were investigated using radioisotope flux techniques.

While the characteristics of transport of putrescine into infected erythrocytes (iRBC) were similar to those of transport into uninfected erythrocytes (RBC) spermidine uptake occurred via the new permeability pathways (NPP) induced by the parasite in the erythrocyte membrane.

Once inside the erythrocyte cytoplasm, both putrescine and spermidine were taken up by the parasite via a temperature- and glucose-dependent, saturable process. The uptake of both these polyamines was competed for by other polyamines, and biosynthesis inhibition led to increased total uptake of both putrescine and spermidine.

Polyamine uptake was pH dependent with uptake increasing with increasing pH, but did not appear to be coupled to the Na+ or K+ gradients. Membrane potential perturbations influenced polyamine uptake, consistent with the transport being an electrogenic process.

